# A Questionnaire Study to Assess Patients With Cleft Lip and Palate for Their Oral Health-Related Quality of Life

**DOI:** 10.7759/cureus.38712

**Published:** 2023-05-08

**Authors:** Amiya Ranjan Sahoo, Shiras Singh Dheer, Mahesh P. C., Pragya Goyal, Ruhi Sidhu, S. Deepalakshmi

**Affiliations:** 1 Department of Oral and Maxillofacial Surgery, Awadh Dental College and Hospital, Jamshedpur, IND; 2 Department of Dentistry, Shrimant Rajmata Vijayaraje Scindia Medical College, Shivpuri, IND; 3 Department of Public Health Dentistry, Dayananda Sagar College of Dental Sciences, Bangalore, IND; 4 Department of Oral Medicine and Radiology, KD Dental College and Hospital, Mathura, IND; 5 Department of Oral Medicine and Radiology, MNDAV Dental College and Hospital, Solan, IND; 6 Department of Oral Medicine and Radiology, Seema Dental College and Hospital, Rishikesh, IND

**Keywords:** children, oral health-related quality of life, questionnaire, cleft palate, cleft lip

## Abstract

Aim: The purpose of this survey was to assess the quality of life of patients with cleft lips and palates in relation to their dental health.

Materials and methods: Between January 2022 and December 2022, 50 people between the age of eight and 15 years who had treatment for cleft lip and/or palate were part of a study. A questionnaire was administered to the subjects, including questions pertaining to their general well-being and dental hygiene. The information was gathered and subjected to statistical analysis through appropriate software, with the outcomes presented in the form of descriptive statistics.

Result:The results of the research showed that those with cleft lip and palate had a significant negative effect on their oral health-related quality of life (OHRQoL). The patients reported having trouble speaking, eating, and smiling, which caused them to feel self-conscious and isolated from other people.

Conclusion: The study's findings show that those born with cleft lip and/or palate have far greater challenges in achieving and maintaining optimal oral health and a satisfying quality of life, which has repercussions for their overall health and happiness. The study's results may provide successful strategies for enhancing patients' OHRQoL who have had treatment for cleft lip and/or palate.

## Introduction

The most common facial birth abnormality is a cleft lip and/or palate (CL/P). The global incidence rate of CL/P is 0.8 per 1,000 live births (World Health Organization, Human Genetics Programme, 2002) [1]. Geographic, racial/ethnic, and gender/sexuality distinctions all have a role in the vast range of its prevalence over the globe [1]. Every point in development at which normal processes are disrupted has the potential to produce a cleft.

CL/P repair is a lengthy process that spans from infancy through adolescence and even young adulthood because its treatment aims to promote normal facial development and growth, achieve full closure of the orofacial cleft, and improve the patient's ability to communicate and interact with others through improved speech, hearing, and facial appearance [2,3]. 

To assess how patients' Oral Health-related Quality of Life (OHRQoL) is connected to their satisfaction with therapy and treatment results. When it comes to individuals with cleft lip and/or palate, it is important to know whether the recommended order of therapy results in a positive OHRQoL. Definitive results are often readily apparent during early adulthood due to maturation, growth factors, and adherence to treatment sequences. The ultimate aim of therapy for CL/P patients, in conjunction with their parents, is to improve the patients' and parents' psychological and social well-being [4]. 

Due to the cumulative impact on a person's well-being, team members must take into account a patient's and even their parents' background, cultural upbringing, current or previous experiences with oral disease and healthcare, current state of mind, expectations for treatment and outcomes, as well as hopes for the future, when providing comprehensive CL/P care. The main objective of treatment for people with CL/P is to ensure their psychological and social well-being, as well as the well-being of their parents. Consider the patient as a whole rather than just the area being treated. Better outcomes and the best possible teamwork among the clinicians will result from an understanding of the full range of complex needs of the patients and parents. 

Compared to patients with higher positive OHRQoL ratings, those with higher negative OHRQoL scores were more likely to want more treatment. The functional and psychosocial effects of oral diseases and disorders are shown by the measurement of oral health-related quality of life. We are asking patients in our clinic who have been diagnosed with CL/P and are getting care from us about their OHRQoL. CL/P have big effects on the lives of the people who have them, their families, and their communities. It is important to learn more about these effects so that effective policies can be made to reduce them. 

## Materials and methods

Researchers from Shrimant Rajmata Vijayaraje Scindia Medical College used a case-control design to do a cross-sectional study on matching between January 2022 and December 2022. The Institutional Review Board has given its approval to the study's protocol with the number IEC/SRVSMC/2022/51. Patients who have had treatment for cleft lip and/or palate were asked to fill out a questionnaire on their dental health and quality of life, measuring how their oral health affected their overall well-being.

Fifty patients between the ages of eight and 15 were chosen for the trial and had surgery to treat their CL/P. Furthermore, 50 patients aged eight to 15 who were CL/P-free served as a comparison group. Patients with a history of CL/P and healthy participants of the same age were randomly assigned to separate research groups with the goal of creating a balanced sample.

Inclusion criteria include patients aged eight to 15 years, with both unilateral and bilateral CL/P, with ASA I status and who wanted to take part in the research and gave written permission after being told about it and their parents also had to give written permission after being told about it. Exclusion criteria include patients with a disability, a syndromic CL/P, or with a mental problem. Students in the control group came from several public and private institutions in Shivpuri, India, and were chosen at random. As such, caregivers comprised the study's third group. Parents and other caretakers of cleft patients agreed to take part in the research. The Child Oral Health Impact Profile (COHIP) was rewarded for carers, and it was filled out by the person who accompanied the patient who had oro-facial clefts.

Totaling 34 items over five categories, the COHIP instrument may be used to measure positive oral, mental, social, functional, and aesthetic states, as well as positive perceptions of oneself and one's educational setting. Using a Likert-type scale with 5 points (from 0 to 4), we've assigned values to each item: 4 for never, 3 for almost never, 2 for sometimes, 1 for fairly often, and 0 for always. If a question is asked and no one answers it, that question receives a score of 0. Scores on this scale range from 0 to 136; an overall better quality of life in terms of oral health is associated with higher total scores.

The data collected were entered in Microsoft Excel (Redmond, WA, USA) and analyzed using the statistical software package SPSS 17.0 (SPSS Inc., Chicago, IL, USA). The independent t-test was used to check the statistically significant difference between the groups, while differences among the cleft lip, cleft palate, and cleft lip and palate total score and subscores were assessed by using one-way ANOVA with a p<0.05, which was considered to be statistically significant.

## Results

Fifty patients participated; 11 were between the ages of eight and 10, 19 were between the ages of 10 and 12, and 20 were between the ages of 12 and 15 who had clefts. Twenty (35% of the total) had unilateral cleft lip and/or palate; eight (21.0%) had cleft lip alone; four (10.5%) had cleft palate only; and 18 (33.5%) had both cleft lips and/or palates. Distributions of control study participants among age group: 20 (45.5%) participants were in the eight to 10 years, six (10.5%) control participants were in the 10 to 12 years, and 24 (54%) participants were in the 12 to 15 years age group.

When compared to the control group, the cleft patients' COHIP scores were found to be lower across all domains, including self-image, school/work environment, functional well-being, and social and emotional well-being. The study demonstrates that cleft patients' overall quality of life is lower than that of the control group, and the difference is statistically significant (Table 1).

**Table 1:** Comparison of total and subscale scores for cleft and control individuals COHIP: Child Oral Health Impact Profile

	Control (n=50)			Cleft (n=50)				
	Average of total score	Mean	Standard deviation	Average of total score	Mean	Standard deviation	T value	p value
Total COHIP	5687	95.33	4.34	5011	77.56	13.44	-3.666	0.001
Oral health	1765	22.22	1.33	1367	14.45	3.66	-7.456	0.001
Functional well being	1123	21.22	1.56	1011	13.33	4.56	-5.323	0.001
Socioemotional wellbeing	1567	34.34	3.45	1187	18.45	7.67	-5.656	0.001
School /work environment	866	13.33	1.88	567	10.43	2.56	-6.454	0.001
Self image	201	2.45	1.44	511	9.56	1.67	11.676	0.001

The group with cleft lips (alveolus) fared best on the COHIP measures of functional and social well-being and the overall score, whereas those with cleft lips and palates (both sides) fared the worst. This shows that the quality of life linked with dental health is best for those with cleft lip (alveolus) compared to the other types of cleft lip and palate, and is at its lowest for those with bilateral cleft lip and palate (Table 2).

**Table 2:** Comparison of individuals' total and subscale scores between cleft groups SD: Standard deviation COHIP: Child Oral Health Impact Profile UCLP: Unilateral cleft lip and palate BCLP: Bilateral cleft lip and palate CL: Cleft lip CP: Cleft palate

	UCLP (N=20)			BCLP (N=18)			CL (N=8)			CP (N=4)				
	Average of total score	Mean	SD	Average of total score	Mean	SD	Average of total score	Mean	SD	Average of total score	Mean	SD	F value	P value
Total COHIP	1675	80.33	18.33	1456	66.45	15.45	110.1	88.12	13.29	712	88.23	13.45	2.343	0.01
Oral health	478	21.22	2.56	412	21.12	2.56	287	22.81	6.86	167	18.66	1.84	1.123	0.41
Functional well being	289	15.67	3.24	205	11.45	5.76	212	20.45	3.64	145	16.45	2.85	2.345	0.02
Socioemotional wellbeing	376	18.22	7.45	334	15.56	9.22	245	25.09	5.62	198	22.23	4.44	2.231	0.04
School /work environment	221	10.34	3.34	198	9.1=87	2.45	134	13.27	2.83	89	10.23	1.34	1.787	0.21
Self image	201	10.22	1.45	234	9.45	2.18	133	12.63	1.74	88	10.45	2.56	1.222	0.28

Caretakers responses indicate that they believe their relations overall quality of life is better, and their COHIP scores were higher than those of patients with cleft patients. Even the subdomains of oral health, functional well-being, social and emotional well-being, school/work environment, and self-image are better in the carers group than the cleft group, as evidenced by higher scores in these categories. The only difference that is not significant is between the school and work environments (p=0.345) (Table 3).

**Table 3:** Comparison of cleft patients and caretakers ratings on many scales COHIP: Child Oral Health Impact Profile

	Cleft (n=50)			Caretaker (n=50)				
	Average of total score	Mean	Standard deviation	Average of total score	Mean	Standard deviation	T value	p value
Total COHIP	5011	77.56	13.44	5123	87.51	13.98	-3.456	0.000
0ral health	1367	14.45	3.66	1456	20.49	4.01	-4.115	0.000
Functional well being	1011	13.33	4.56	1135	14.01	5.45	-2.065	0.031
Socioemotional wellbeing	1187	18.45	7.67	1356	19.61	8.91	-2.156	0.039
School /work environment	567	10.43	2.56	645	11.62	3.05	-0.645	0.345
Self image	511	9.56	1.67	598	11.5	2.65	-3.987	0.001

The treatment and satisfaction with various aspects of the form and function of the face were not significantly impacted by the cleft patients or caretaker replies. The features of the face, the appearance of the nose, and the appearance of the lip leave both cleft lip and palate patients and their carers the least content (Table 4).

**Table 4:** Comparison of the cleft assessment profiles of patients with clefts and their carers

	Cleft (n=50)			Caretaker (n=50)				
	Average of total score	Mean	Standard deviation	Average of total score	Mean	Standard deviation	T value	p value
Speech	135	2.05	1.61	150	2.78	1.75	-1.009	0.320
Hearing	291	3.51	0.31	298	3.91	0.46	-1.005	0.301
Appearance of teeth	145	2.97	1.30	151	3.01	1.39	-1.407	0.145
Appearance of nose	103	1.61	1.29	119	1.80	1.35	-1.356	0.192
Breathing through nose	141	2.20	1.74	156	2.45	1.81	-1.120	0.248
Appearance of lip	98	1.55	1.30	112	1.71	1.49	-0.697	0.518
Profile of face	106	1.8	1.41	119	2.1	1.56	-0.545	0.594

## Discussion

Quality of life (QoL) is significantly impacted by CL/P in afflicted children. Measuring QoL is crucial for those with craniofacial deformities like CL/P [5,6], which persists sadly into the new century. A number of research teams have written concerning the development of QoL instruments, but only one has produced an instrument that has been "internationally certified" to measure children's quality of life [7]. To assess children's oral health-related QoL, researchers developed the COHIP as part of a large-scale, global investigation. Broder and Wilson-Genderson adapted and validated the COHIP for use in the English-speaking world so that it could be used to evaluate how someone felt about their oral health, their ability to go about their daily activities, their social and emotional life at school, and their overall sense of self-worth [8]. To assess how cleft lip and/or cleft palate affect a child's quality of life, Broder et al. created a questionnaire [6]. It was found that the scale had a high level of internal consistency and strong test-retest reliability.

Pedro et al. discovered that individuals with CL/P had a worse quality of life when they had impairments in their functional well-being or their educational environment [1]. Health-related quality-of-life parameters for cleft patients showed no significant difference by gender, according to an investigation by Andreas et al. [9]. The middle-aged group (11-18 years old) has a lower overall mean compared to the older and younger age groups in various categories. There is no statistical significance between the three age groups. Eslami et al. [10] found that those with a cleft lip or cleft palate did not age into a worsening of their oral health-related quality of life. The results were the same for those under 12 and those over 12 years old. Because there was a notable distinction between cleft kinds. In our study, there are statistically significant variations across cleft types in terms of "functional well-being", "social and emotional well-being," and the total COHIP score (p = 0.002, 0.004, and 0.001, respectively). Those with an alveolar cleft lip scored the highest, whereas those with cleft lips and palates on both sides scored the lowest. It's important to remember that there are many distinct kinds of clefts, and each one calls for a unique treatment plan. In our research, cleft lip (alveolus) patients had the highest scores on the total COHIP score, which is quite close to the result of Bos et al. [11], who demonstrated that this metric significantly differentiated across the different types of clefts. According to research conducted by Eslami et al. [10], using the COHIP questionnaire, researchers discovered no statistically significant difference between the impacts of unilateral and bilateral clefts on oral health-related quality of life. Direct feedback from cleft patients, rather than their carers, is always desired. Our findings are consistent with those of research by Bos et al. [11] using COHIP, which also found that kids outscore their parents.

When it came to parental ratings, we found that lips received the lowest average score, followed by noses, teeth, and overall facial profiles. The patients' mean score for their teeth was the lowest of all the categories tested, followed by their lips, their speech, and their nose. The cleft group and the caretakers group are similar in every way that matters. The lowest possible score indicates the least amount of contentment from both the patient and the caretaker. This doesn't rule out our study's final hypothesis, either. It was expected that parents of children with unrepaired orofacial clefts and those of children who had their clefts corrected would not report any significant differences in their children's levels of happiness with different facial features.

Because of how prominent the teeth and lips are in the overall aesthetic, people with dental abnormalities may be harassed or assaulted because of their look. Treating children with dental malformations in a multidisciplinary manner and referring them to the appropriate doctors is crucial. Cleft lip and/or cleft palate malformations often cause speech and hearing impairments, which may be a hindrance to effective communication [12,13]. Children with cleft palate, who often have severe speech impairments and other learning problems, require regular monitoring and intensive treatment not just for cleft-related disorders but also for the potential for other developmental issues, such as social and emotional difficulties. A variety of variables may contribute to cleft lip and palate development in various populations (e.g., speech, face, adjustment, and learning). Patients who express greater dissatisfaction with their voice or appearance should be offered counseling and more intensive clinical services [14-16] to help them work through their issues and improve their communication.

The study's limitations include a smaller sample size and the disparities that result from the wide age range of patients who were included, the type of the clefts, and the study's intervention period that need to be examined in further longitudinal studies for improved outcomes.

## Conclusions

A cleft lip and palate condition hampers life and has an adverse impact on the functionality and emotions of the person affected. Oral health maintenance is a big challenge for these people. The participants with cleft had lower oral health-related quality of life compared with healthy participants in all domains. The caretaker's perceptions of their child's oral health-related quality of life were higher than those of their children with repaired orofacial clefts. Between different types of CL/P, the carer's perceptions of their child's oral health-related quality of life differ significantly, particularly in terms of functional well-being, socio-emotional well-being, and overall oral health quality of life. 

Those with cleft lips (alveolus) had the highest quality of life in terms of oral health, whereas those with clefts on both sides of the mouth had the lowest. The perceptions of the carers of their children's contentment with different parts of their faces were the same as those of their children with repaired orofacial clefts. Both cleft lip and palate patients and their carers are least satisfied with the features of the face, the appearance of the nose, and the appearance of the lip.
